# 2019 GSA FELLOWS SYMPOSIUM: STRENGTH IN AGE: HARNESSING THE POWER OF NETWORKS

**DOI:** 10.1093/geroni/igz038.1978

**Published:** 2019-11-08

**Authors:** Peter Martin

**Affiliations:** Iowa State University, Ames, Iowa, United States

## Abstract

The 2019 GSA Fellows Symposium includes GSA members who were recently granted GSA Fellow status. They represent each of the GSA sections: Biological Sciences (BS), Health Sciences (HS), Behavioral & Social Sciences (BSS), Social Research, Policy & Practice (SRPP), and the Academy for Gerontology in Higher Education (AGHE). The theme of the 2019 GSA Fellows Symposium is focusing on the power of networks and will highlight the importance of neuroendocrine networks (Christian Sell), the role of social support for refugees in Canada (Esme Fuller-Thomson), the importance of professional partnerships to promote health (Heather Young), aging-friendly communities (Emily Greenfield), and the role of networks in teaching gerontology (Tina Kruger). The importance of networks across disciplinary boundaries will be discussed.

